# The PlantLIBRA consumer survey: Findings on the use of plant food supplements in Italy

**DOI:** 10.1371/journal.pone.0190915

**Published:** 2018-01-11

**Authors:** Patrizia Restani, Chiara Di Lorenzo, Alicia Garcia-Alvarez, Gianfranco Frigerio, Francesca Colombo, Franco M. Maggi, Raimon Milà-Villarroel, Lluis Serra-Majem

**Affiliations:** 1 Dept. of Pharmacological and Biomolecular Sciences, Università degli Studi di Milano, Milano, Italy; 2 Fundación para la Investigacion Nutricional, Barcelona Science Park, University of Barcelona, Barcelona, Spain; 3 Ciber Obn Fisiopatologıa de la Obesidad y la Nutrición, Instituto de Salud Carlos III, Madrid, Spain; 4 Institute of Biomedical and Health Research of Las Palmas, University of Las Palmas de Gran Canaria, Las Palmas de Gran Canaria, Spain; Defence Research and Development Organisation, INDIA

## Abstract

**Background:**

Food supplements, and in particular those containing botanicals (plant food supplements, PFS), have in recent decades been of great interest both to consumers and to food/pharmaceutical industries.

**Objectives:**

The aim of this paper is to examine replies by Italian consumers to the PlantLIBRA consumers' survey in order to: 1) assess the behaviour of an Italian population with respect to the use of PFS, and to compare it with that of other 5 countries involved in the whole survey; 2) identify different habits in the 4 Italian cities selected according to their geographical distribution; 3) collect independent information on the actual intake of PFS and consumers' behaviour.

**Subjects/setting:**

397 Italian consumers enrolled, 187 males (49.5%) and 191 female (50.5%). The distribution of subjects among the 4 cities included was: Milan 99; Venice 90; Rome 96 and Catania 96.

**Results:**

The interest in PFS in Italy is high, the prevalence of "regular" consumers being 22.7%. Some differences were observed between the 4 cities involved: the pattern of use during the year was specific to each city; consumers in Milan reported reasons to use PFS significantly different from those in the whole Italian sample and did not indicate supermarkets as an important place of purchase; respondents from Rome and Catania more frequently used family doctors and pharmacists as a source of recommendation. Some significant difference among cities, sex and age groups were observed when the most frequently used botanicals were ranked.

**Conclusions:**

The results provide new insights on the socio-economic characteristics and lifestyle of Italian PFS consumers, on their reasons for and pattern of use, and on their behaviour and expectations. The value of this information is not restricted to the specific country (Italy) but allows for a more general evaluation of the pattern of use, according to habits and geographical area.

## Introduction

Food supplements, especially those containing botanicals, named plant food supplements (PFS), have been of increasing interest in recent decades to consumers and food/pharmaceutical industries [1–2]. The relative market has greatly increased in all five continents, becoming an important economic business in the area of human health.

The range of products presented in different distribution channels (supermarkets, pharmacy, herbal shops, internet) and their self-prescription raised concerns about the balance between risk and benefit [3–4], and some research projects have been funded to produce reliable data on this topic. Among the others, a European project called PlantLIBRA (Plant Food Supplements: Levels of Intake, Benefit and Risk Assessment) was financed within the 7th Framework Programme under grant agreement n. 245199.

The first consideration was the classification (and selection for survey) of categories where botanicals can be present as ingredients: i.e. foods, food supplements, herbal medicinal products (traditional medicine), homeopathic products, cosmetics, etc. In Italy, as well as in European Union (EU), most products containing botanicals are sold as food supplements and regulated under the food law [5–6]; as defined by the project, the category of Plant Food Supplements (PFS) was the only one included in the recruitment of consumers.

In agreement with EFSA, in the PlantLIBRA Consumer Survey, "Botanical" meant either raw material or derived preparations made from plants, algae, fungi or lichens (http://www.efsa.europa.eu/en/topics/topic/botanicals). The botanicals to be included in the survey were clearly defined at the outset; PFS were defined as the "*foodstuffs the purpose of which is to supplement the normal diet and which are concentrated sources of botanical preparations that have nutritional or physiological effect*, *alone or in combination with vitamins*, *minerals and other substances which are not plant-based*". Herbal remedies, other medicinal products based on botanicals, herbal teas and juices were excluded [7].

Survey data collection and the main objectives of the consumer survey have been described in a previous paper by Garcia-Alvarez et al [7]. The main goals of this paper were: 1) the assessment of the pattern of PFS use in Italy in comparison with the other 5 European countries (Finland, Germany, Romania, Spain and the United Kingdom) involved in the whole survey; 2) the identification of different consumption habits in the 4 Italian cities selected according to their geographical distribution (Milan, Venice, Rome and Catania); and 3) the collection of information to verify the actual intake of PFS and consumers' behaviour.

## Materials and methods

The PlantLIBRA Consumer Survey was conducted in 6 European countries (Finland, Germany, Italy, Romania, Spain and the United Kingdom), and recruitment of participants occurred in 4 cities for each country. In Italy, the cities included were selected as a reference for different geographical areas: Milan for northern region, Venice for northern-eastern region; Rome for central region, and Catania for southern region/islands.

In order to obtain a sample of approximately 400 Italian consumers (as established), 1951 individuals were pre-screened [7]. Consumers were eligible if in the previous 12 months their PFS consumption was at least 1) one daily dose for at least 2 consecutive or non-consecutives weeks; or 2) one or more doses per week for at least 3 consecutive weeks; 3) one or more doses per week for at least 4 non-consecutive weeks.

Eligible consumers completed a detailed questionnaire on PFS usage, providing product/plant names, form of dosage, frequency of use, reasons for use, adverse effects, places and patterns of purchase and information sources on products. Data on a maximum of five different PFS for each consumer was recorded; when PFS were more than 5, their inclusion was based on the frequency of use. Responders' socio-demographic data, including age, gender, level of education and employment status, as well as height, weight and health-related lifestyle information, were also collected. Further details on the survey have been published previously [7]. The composition of each PFS was obtained from the label, if available or, when only the name was known, by searching the PFS ingredients in the producers’ website.

## Ethical aspects

Approval of the survey protocols was obtained from the Ethics Committee of the Università degli Studi di Milano, Italy. The approval required submitting all survey material to the members for evaluation. Furthermore, the ethical aspects were considered in the European Commission Consolidated Review Report dated 30^th^ September 2013 and evaluated as “*ethical issues regarding the surveys have been handled appropriately*”.

Informed consent was obtained from survey participants verbally after they had read the survey information sheet. The data were collected anonymously on paper questionnaires and then transferred to an electronic database; all respondents were assigned an ID number prior to data analyses.

## Statistical analysis

All data were entered into the statistical package Statistical Package for Social Science (SPSS) for Windows v. 18 (IBM Corporation, Somers, NY, USA), which was used for analysis. Respondent data were recorded in a separate database and a number of variables were created and/or recoded to facilitate reporting and analysis.

Absolute frequencies and percentages for each of the variable categories were used to describe the qualitative nominal/ordinal and discrete quantitative survey data. In turn, all data have been stratified by gender, age range and country—also using absolute frequencies and percentages and 95% confidence intervals. When describing the association between two qualitative variables (nominal or ordinal), contingency tables were used. The continuous quantitative variables (e.g. BMI, alcohol) were recoded into categorical variables. For details about the statistical analysis and data organization see the paper by Garcia-Alvarez et al [7].

## Results

### Description of the consumer sample

Data included in this paper were collected during the main survey of the European Project PlantLIBRA and are here focused on the behaviour and perception of Italian PFS consumers.

The PlantLIBRA Consumer Survey included approximately 400 consumers for each country (Finland, Germany, Italy, Romania, Spain, UK) for a total of 2359 individuals. In Italy, the consumers enrolled were 378, 187 males (49.5%) and 191 female (50.5%). Details about sample distribution among the 4 Italian cities are reported in Table 1. The population was ranked in two age groups: 1) 18–59 years including 284 people (75.1%) and 2) >60 years including 94 consumers (24.9%). The distribution of consumers by sex and age groups was established, as inclusion criteria, in the survey protocol [7]. As for the European sample (ES), the high school was the educational level (medium) reached by the majority of Italian consumers involved in the survey (58.7% vs 65.7% of the ES); the lowest level (primary school) was attained by 19.1% of the Italian and 10.6% of the ES. The distribution was similar at local level, and the percentage of people who reached the medium education level ranged between 52.1% (Catania) and 68.8% (Rome). Catania was the city with the highest percentage of graduate consumers enrolled (36.5%).

**Table 1:** PlantLIBRA Italian consumer survey sample—socio-economic characteristics, overall and by city.

Characteristic	All countries		Italy		Milan		Venice		Rome		Catania	
	n	%	n	%	n	%	n	%	n	%	n	%
**Consumer sample**												
Total	2359	100	378	100	96	100	90	100	96	100	96	100
Male	1141	48.4	187	49.5	39	40.6	46	51.1	52	54.2	50	52.1
Female	1218	51.6	191	50.5	57	59.4	44	48.9	44	45.8	46	47.9
**Age**												
m±SD	46.4±15.6		44.0±16.2		46.8±15.3		40.43±16.2		45.8±15.7		42.8±17.2	
18–59 years	1764	74.8	284	75.1	69	71.9	73	81.1	71	74.0	71	74.0
> 60 years	595	25.2	94	24.9	27	28.1	17	18.9	25	26.0	25	26.0
**Education**^**a**^												
Low	249	10.6	72	19.1	25	26.0	22	24.4	14	14.6	11	11.5
Medium	1549	65.7	222	58.7	54	56.3	52	57.8	66	68.8	50	52.1
High	561	23.8	84	22.2	17	17.7	16	17.8	16	16.7	35	36.5
**Employment status**												
Employed	1357	57.5	221	58.5	61	63.5	46	51.1	61	63.5	53	55.2
Retired	498	21.1	50	13.2	13	13.5	10	11.1	15	15.6	12	12.5
Student	187	7.9	47	12.4	5	5.2	14	15.6	12	12.5	16	16.7
Housework	157	6.7	39	10.3	10	10.4	15	16.7	6	6.3	8	8.3
Unemployed	142	6.0	21	5.6	7	7.3	5	5.6	2	2.1	7	7.3
Other	18	0.8	0	0	0	0	0	0	0	0	0	0

^a^ Low education level = Primary school; Medium education level = High School; High education level = Graduation

Most of the Italian consumers included were employed (58.5 vs 57.5% of ES); compared to the European one, the Italian sample included fewer retired people (13.2 vs. 21.1%), while students and people doing housework were more represented. Although with some variability for the percentage of students and unemployed persons, the distribution of consumers of the 4 Italian cities among the employment status was similar.

Overall and for the 4 cities, approximately 70% of Italian consumers declared their health status as good or very good (Table 2); only two respondents reported it bad (nobody very bad), both located in Catania. Most Italian respondents (87.8%) declared a low/moderate amount of physical activity (Table 2), whereas in the European sample as a whole (ES) it was moderate/high (81.5%). No significant difference was observed between Italian cities. Despite the lower physical activity, 65.1% of Italian respondents reported a BMI (Body Mass Index) as normal (18.5–25.0 kg/m^2^), while only 47.1% of the ES fell into this category, and only 5.8% of the Italian sample was obese, versus 15.1% in the ES.

**Table 2:** PlantLIBRA Italian PFS consumer survey—heath-related lifestyle sample characteristics. Overall and by city.

Characteristic	All countries		Italy		Milan		Venice		Rome		Catania	
	n	%	n	%	n	%	n	%	n	%	n	%
**Self-reported health status**												
Very good	353	15.0	22	5.8	1	1.0	2	2.2	10	10.4	9	9.4
Good	1427	60.5	243	64.3	68	70.8	62	68.9	54	56.3	59	61.5
Neither bad nor good	496	21.0	111	29.4	27	28.1	26	28.9	32	33.3	26	27.1
Bad	70	3.0	2	0.5	0	0	0	0	0	0	2	2.1
Very bad	13	0.6	0	0	0	0	0	0	0	0	0	0
**BMI**^**a**^												
Underweight (<18.5 kg/m^2^)	69	2.9	12	3.2	3	3.1	2	2.2	5	5.2	2	2.1
Normal weight (18.5–25.0 kg/m^2^)	1116	47.3	246	65.1	63	65.6	55	61.1	66	68.8	62	64.6
Overweight (25.0–30.0 kg/m^2^)	818	34.7	98	25.9	25	26.0	25	27.8	21	21.9	27	28.1
Obesity (>30.0 kg/m^2^)	356	15.1	22	5.8	5	5.2	8	8.9	4	4.2	5	5.2
**Physical activity**^**b**^^,^^**c**^												
Low	436	18.5	141	37.3	46	47.9	47	52.2	24	25.0	24	25.0
Moderate	909	38.6	191	50.5	38	39.6	36	40.0	65	67.7	52	54.2
High	1012	42.9	45	11.9	12	12.5	6	6.7	7	7.3	20	20.8
**Smoking habit**												
Never smoker	1100	46.6	181	47.9	52	54.2	44	48.9	36	37.5	49	51.0
Former smoker	544	23.1	85	22.5	16	16.7	13	14.4	33	34.4	22	22.9
Smoker	715	30.3	112	29.6	28	29.2	33	36.7	27	28.1	25	26.0
**Alcohol consumption**												
< 1 time/day	1398	59.3	116	30.7	17	17.7	35	38.9	30	31.2	35	36.5
≥ 1 time/day	296	12.6	156	41.3	51	53.1	40	44.4	36	37.5	28	29.2
Uncertain	665	26.0	106	28.0	28	29.2	15	16.7	30	31.2	33	34.4
**Regular use of food supplements (excluding PFS)**												
Yes	767	32.5	63	16.7	15	15.6	16	17.8	25	26.0	19	19.8
No	1536	65.1	311	82.3	80	83.3	72	80.0	70	72.9	77	80.2
Uncertain	56	2.4	4	1.1	1	1.0	2	2.2	1	1.1	0	0
**CAM**^**d**^ **Usage**												
Yes	947	40.1	96	25.4	25	26.0	24	26.6	21	21.8	26	27.0
No	1412	59.9	282	74.6	71	73.9	66	73.3	75	78.1	70	72.9
***Total sample***	*n = 2359*		*n = 378*		*n = 96*		*n = 90*		*n = 96*		*n = 96*	

^a^BMI = Body Mass Index: weight (kg)/heigh (m^2^)

^b^Two consumers, one from Germany and one from Italy/Venise, did not reply to this question

^c^IPAQ categories [8]

^d^CAM = Complementary and Alternative Medicine (acupunture, chiropractor, massage therapist, etc.)

Most Italian consumers were non-smokers and approximately 50% of them had never smoked (Table 2). A significant percentage (41.3%) of Italian consumers declared a consumption of an alcoholic beverage more than once a day, compared to the ES (12.6%). The difference observed with the whole survey was probably due to the fact that consumption of wine during meals is considered positively by Italian society and there is consequently no reluctance in declaring it. The large majority of Italians do not use food supplements other than PFS, or complementary and alternative medicines (CAM) such as acupuncture, chiropractic, or massage therapy.

### Pattern of PFS consumption in Italy

Similarly to the ES, more than 90% of the Italian subjects consumed one PFS, and only 0.8% (ES 4%) more than two PFS (Table 3). The number of consumers taking one PFS was higher in Central/Southern Italy (Rome 97.9% and Catania 93.8%), where no respondent declared the use of more than two PFS. As in the whole survey, solid forms (capsules, pills, tablets, lozenges) were the most frequently used in Italy, followed by liquid forms, which were more often cited by Venice respondents. Regarding the pattern of use (Table 3), 70% of Italian respondents claimed to use PFS only periodically or when their health status worsened; use "whenever/sporadically" was fairly frequently cited by consumers from Milan (30.2%) and rarely by those from Rome (2%).

**Table 3:** PlantLIBRA Italian PFS consumer survey—PFS usage pattern and form, overall and by city.

Characteristic	All countries		Italy		Milan		Venice		Rome		Catania	
	n	%	n	%	n	%	n	%	n	%	n	%
**Number of PFS used**	*n = 2359*		*n = 378*		*n = 96*		*n = 90*		*n = 96*		*n = 96*	
1	1 975	87	343	90.7	76	79.2	79	87.8	94	97.9	90	93.8
2	289	12.3	32	8.5	16	16.7	11	12.2	2	2.1	6	6.3
3–5	95	4.0	3	0.8	4	4.2	0	0	0	0	0	0
**Form of PFS**^**a**^	*n = 2874*		*n = 417*		*n = 116*		*n = 101*		*n = 98*		*n = 102*	
Capsules	1101	38.3	144	34.5	46	39.7	32	31.7	36	36.7	30	29.7
Pills/Tablets/Lozenges	1057	36.8	126	30.2	29	25.0	25	24.8	33	33.7	39	38.6
Liquid	513	17.9	110	26.4	33	28.4	34	33.7	23	23.5	20	19.8
Ampoules	104	3.6	13	3.1	2	1.7	3	3.0	2	2.0	6	5.9
Other	99	3.4	24	5.7	6	5.2	7	6.9	4	4.1	6	5.9
**Pattern of use^**	*n = 2874*		*n = 417*		*n = 116*		*n = 101*		*n = 98*		*n = 102*	
Whenever/sporadically	568	19.8	73	17.5	35	30.2	15	14.9	2	2.0	21	20.6
Periodically	1072	37.3	172	41.3	33	28.4	45	44.6	54	55.1	40	39.2
Worsening health status	638	22.2	128	30.7	37	31.9	38	37.6	20	20.4	33	32.3
Other reasons	512	17.8	32	7.7	11	9.5	3	3.0	12	12.2	6	5.9
Uncertain	84	2.9	12	2.9	0	0	0	0	10	10.2	2	2.0

^a^Numbers and percentages are referred to the total PFS used and not to the total consumer samples

**Fig 1:**
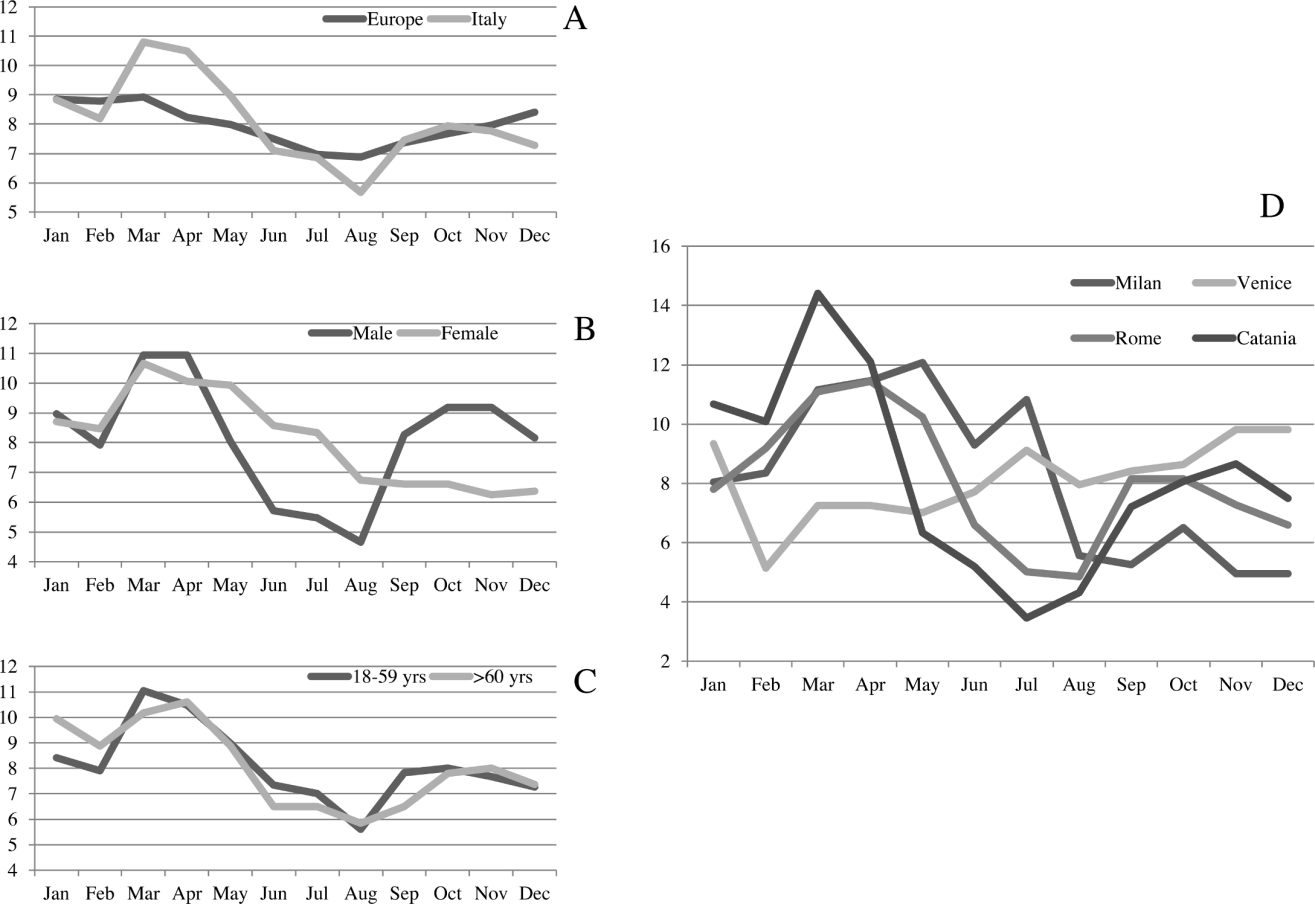
Pattern of PFS use during the year. (A) Italian sample versus whole survey sample; (B) male versus females; (C) younger versus older respondents; (D) data from the 4 Italian cities.

The percentage of consumers using PFS all the year round was low in the whole survey (4.4% of the ES and 2.6% of the Italian respondents), but the pattern of use during the year showed several interesting differences (Fig 1). While the ES showed a quite constant pattern of use during the year, Italian consumers increased their use of PFS in spring and reduced it in summer; this pattern is particularly evident for males, while no important difference was seen between the two age groups. The pattern of consumption differs among the four Italian cities, with opposite profiles when Venice and Catania are considered. These data suggest that geographical area and climatic conditions can modulate the habits of PFS consumers.

With regard to the reasons of use (Table 4), the three most reported items by Italian respondents were, in decreasing order of importance: 1) stomach/digestive function; 2) energy and tonics; and 3) relaxing; the last item was less cited by ES in favour of ‘boosting the immune system’. The ranking of reasons for use in Rome and Catania was similar to the average for all Italian respondents, but for Milan the three most important items were: 1) digestive function, 2) immune system, and 3) hair/skin; and for Venice 1) digestive function, 2) energy/tonics, and 3) body weight control. More details on reasons of use in the whole survey (6 countries), in Italy, and in the four cities are listed in Table 4. The difference between the number of reasons for use of PFS given by the responders (in Italy 417) and the number of total counts (549) shows that, generally speaking, more than one reason of use was reported for most PFS.

**Table 4:** Reasons of use, overall and by city^a^^,^^b^^,^^c^.

	Europe		Italy		Milan		Venice		Rome		Catania	
	n	%	n	%	n	%	n	%	n	%	n	%
Stomach/Digestive function	386	13.4	80	19.2	24	20.7	20	19.8	17	17.3	19	18.6
Energy/Tonics	480	16.7	61	14.6	19	16.4	10	9.9	15	15.3	17	16.7
Relaxing	266	9.3	48	11.5	9	7.8	9	8.9	13	13.3	17	16.7
Immune system	578	20.1	38	9.1	17	14.7	7	6.9	5	5.1	9	8.8
Body weight	253	8.8	34	8.2	10	8.6	14	13.9	5	5.1	5	4.9
Sleeping	196	6.8	33	7.9	10	8.6	4	4.0	8	8.2	11	10.8
Hair/skin	309	10.8	32	7.7	15	12.9	7	6.9	4	4.1	6	5.9
Hearth/blood circulation	223	7.8	27	6.5	12	10.3	7	6.9	6	6.1	2	2.0
Joint and bones	258	9.0	26	6.2	8	6.9	2	2.0	12	12.2	4	3.9
Flu/cold	310	10.8	24	5.8	6	5.2	7	6.9	4	4.1	7	6.9
Mood	206	7.2	24	5.8	7	6.0	8	7.9	4	4.1	5	4.9
Antioxidant intake	189	6.6	18	4.3	11	9.5	2	2.0	4	4.1	1	1.0
Memory	236	8.2	17	4.1	5	4.3	5	5.0	4	4.1	3	2.9
Cholesterol	164	5.7	16	3.8	3	2.6	3	3.0	4	4.1	6	5.9
Urinary tract	137	4.8	14	3.4	5	4.3	4	4.0	1	1.0	4	3.9
Vision or hearing	67	2.3	6	1.4	2	1.7	0	0.0	3	3.1	1	1.0
Menopause	168	5.8	5	1.2	4	3.4	0	0.0	0	0.0	1	1.0
General health	84	2.9	1	0.2	2	1.7	3	3.0	5	5.1	3	2.9
Other	265	9.2	45	10.8	11	9.5	10	9.9	16	16.3	8	7.8
Uncertain	9	0.3	0	0.0	0	0.0	0	0.0	0	0.0	0	0.0
*Total PFS used*	*n = 2874*		*n = 417*		*n = 116*		*n = 101*		*n = 98*		*n = 102*	
*Total counts of reasons*	*n = 4784*		*n = 549*		*n = 180*		*n = 122*		*n = 130*		*n = 129*	
*Total counts/Total PFS*	*1*.*7*		*1*.*3*		*1*.*6*		*1*.*2*		*1*.*3*		*1*.*3*	

^a^grey cells indicate the three most reported reasons of use

^b^the consumer could indicate for each PFS several reasons of use; percentage are referred to the total PFS used

^c^reasons of use are listed according to the decreasing order of reply coming from the Italian respondents

### Place of purchase and sources of information

In the whole survey and in Italy, the most usual places of purchase were, in decreasing order of importance: 1) health/herbal shops, 2) pharmacy, and 3) supermarket/grocery stores (Table 5). Supermarkets were not as frequently cited by Italian respondents as in the whole survey (7.9% vs. 13.2%), and in Milan was not ranked even at the third position, being cited only three times.

**Table 5:** Places of purchase, overall and by city^a^^,^^b^^,^^c^.

	Europe		Italy		Milan		Venice		Rome		Catania	
	n	%	n	%	n	%	n	%	n	%	n	%
Health/herbal shop	1537	53.5	271	65.0	76	65.5	74	73.3	61	62.2	60	58.8
Pharmacy	781	27.2	99	23.7	28	24.1	13	12.9	24	24.5	34	33.3
Supermarket/grocery store	379	13.2	33	7.9	3	2.6	7	6.9	12	12.2	11	10.8
Internet	197	6.9	13	3.1	4	3.4	3	3.0	1	1.0	5	4.9
Other	186	6.5	10	2.4	5	4.3	1	1.0	2	2.0	2	2.0
Friends/relatives	29	1.0	5	1.2	4	3.4	1	1.0	0	0	0	0
Uncertain	192	6.7	5	1.2	0	0	1	1.0	3	3.1	1	1.0
Gym	36	1.3	3	0.72	2	1.7	1	1.0	0	0	0	0
Total PFS used	*n = 2874*		*n = 417*		*n = 116*		*n = 101*		*n = 98*		*n = 102*	

^a^grey cells indicate the three most reported places of purchase

^b^the consumer could indicate several places of purchase for each PFS; percentages are referred to the total PFS used

^c^places of purchase are listed according to the decreasing order of reply coming from the Italian respondents

As shown in Table 6, the most frequently reported sources of recommendation in Italy were, in decreasing order of citation: 1) herbal shop assistants, 2) friends and relatives, and 3) nobody/myself. Italian consumers cited herbal shop assistants more frequently than the rest of the ES (39.7% vs 15.3%). The special trust in the expertise of these professionals is probably due to the fact that most of them are graduates in "herboristic sciences and technologies", a three-year course offered by the Faculty of Pharmacy. In Rome and Catania, the third item listed was not "nobody/myself" but "pharmacist" and "internet/social groups", respectively.

**Table 6:** Reported sources of recommendation, overall and by city^a^^,^^b^^,^^c^.

	Europe		Italy		Milan		Venice		Rome		Catania	
	n	%	n	%	n	%	n	%	n	%	n	%
Health/herbal shop assistant	440	15.3	145	34.8	46	39.7	37	36.6	29	29.6	33	32.4
Friends/relatives	1066	37.1	128	30.7	38	32.8	28	27.7	31	31.6	31	30.4
Nobody/myself	618	21.5	62	14.9	22	19.0	17	16.8	8	8.2	15	14.7
Pharmacist	279	9.7	56	13.4	12	10.3	8	7.9	16	16.3	18	17.6
Family doctor	302	10.5	52	12.5	8	6.9	5	5.0	13	13.3	26	25.5
Internet/social group	179	6.2	13	3.1	1	0.9	1	1.0	0	0	11	10.8
Homeopath	144	5.0	13	3.1	4	3.4	5	5,0	2	2.0	2	2.0
Magazine/newspaper	220	7.7	7	1.7	1	0.9	2	2.0	1	1.0	3	2.9
Nutritionist/dietician	234	8.1	5	1.2	2	1.7	1	1.0	1	1.0	1	1.0
Paramedic personnel^d^	36	1.3	5	1.2	1	0.9	1	1.0	1	1.0	2	2.0
TV/Radio	59	2.1	4	1.0	0	0	2	2.0	0	0	2	2.0
Gym trainer	8	0.3	4	1.0	2	1.7	0	0	1	1.0	1	1.0
Uncertain	32	1.1	1	0.2	0	0	0	0	1	1.0	0	0
Brochure/Leaflet	37	1.3	0	0	0	0	0	0	0	0	0	0
Books/scientific journals	3	0.1	0	0	0	0	0	0	0	0	0	0
Telemarketing/Network marketing	12	0.4	0	0	0	0	0	0	0	0	0	0
CAM^e^ Therapist	10	0.3	0	0	0	0	0	0	0	0	0	0
Total PFS used	*n = 2874*		*n = 417*		*n = 116*		*n = 101*		*n = 98*		*n = 102*	

^a^grey cells indicate the three most reported sources of recommendation

^b^the consumer could indicate several sources of recommendation for each PFS; percentage are referred to the total PFS used

^c^reported sources of recommendation are listed according to the decreasing order of reply coming from the Italian respondents

^d^including nurses, opticians, physical therapists

^e^CAM = Complementary and Alternative Medicine

### Consumer perception and behaviour

As illustrated in Table 7, Italian respondents believed that consumption of PFS had helped their health always (31%) or sometimes (57%), with an opposite trend in the ES (57% always, 31% sometimes). A certain percentage of consumers (9.4%) was not satisfied with the efficacy of PFS, and replied to the question "*Did the PFS help you*?*"* with the items "rarely" of "not at all". Venice and Catania showed the lowest and the highest number of sceptical consumers, respectively. Most Italian consumers had not informed the family doctor (73.6%) or the pharmacist (63.5%) about their use of PFS despite more than 50% of respondents not feeling well-enough informed. Catania consumers had more frequently informed the family doctor (37.3%) or the pharmacist (48%).

**Table 7:** PlantLIBRA Italian PFS consumer survey—consumers' perception and behaviour, overall and by city.

Question	All countries		Italy		Milan		Venice		Rome		Catania	
	n	%	n	%	n	%	n	%	n	%	n	%
**Did the PFS help you?**												
Always	1623	56.5	128	30.7	30	25.9	38	37.6	31	31.6	29	28.4
Sometimes	884	30.8	236	56.6	68	58.6	56	55.4	57	58.2	55	53.9
Rarely	103	3.6	35	8.4	10	8.6	4	4.0	7	7.1	14	13.7
Not at all	74	2.6	4	1.0	1	0.9	1	1.0	0	0	2	2.0
Uncertain	190	6.6	14	3.4	7	6.0	2	2.0	3	3.1	2	2.0
**Have you informed the family doctor of PFS use?**^**a**^												
Yes	739	25.7	105	25.2	13	11.2	28	27.7	26	26.5	38	37.3
No	2098	73.0	307	73.6	101	87.1	73	72.3	71	72.4	62	60.8
Uncertain	37	1.3	5	1.2	2	1.7	0	0	1	1.0	2	2.0
**Have you informed the pharmacist of PFS use?**^**a**^												
Yes	692	24.1	147	35.3	34	29.3	28	27.7	36	36.7	49	48.0
No	2130	74.1	265	63.5	81	69.8	73	72.3	61	62.2	50	49.0
Uncertain	52	1.8	5	1.2	1	0.9	0	0	1	1.0	3	2.9
**In general, do you feel informed enough on PFS?**^**b**^												
Well-informed	287	12.2	13	3.4	1	1.0	2	2.2	3	3.1	7	7.3
Quite informed	1205	51.1	155	41.0	38	39.6	48	53.3	29	30.2	40	41.7
Not enough	703	29.8	163	43.1	45	46.9	35	38.9	47	49.0	35	37.5
Not at all	106	4.5	35	9.3	12	12.5	4	4.4	10	10.4	9	9.4
Uncertain	58	2.5	12	3.2	0	0	1	1.1	7	7.3	4	4.2
**Have you experienced adverse effects?** ^**b**^												
Yes	82	2.9	5	1.2	2	1.7	0	0	1	1	2	2.0
No	2792	97.1	412	98.8	114	98.3	101	100	97	99	100	98.0
***Total PFS used***	*n = 2874*		*n = 417*		*n = 116*		*n = 101*		*n = 98*		*n = 102*	
***Total consumers' sample***	*n = 2359*		*n = 378*		*n = 96*		*n = 90*		*n = 96*		*n = 96*	

^a^percentages refer to Total PFS used

^b^percentages refer to Total consumers' sample

Only 5 consumers (1.2%) reported adverse effects: 2 from Milan, 1 from Rome and 2 from Catania (Table 7). The cases are summarized below; details and comparison with other countries can be found in the paper by Restani et al. [9]:

1. A consumer with a history of allergic reactions experienced difficulty in swallowing after consumption of a PFS containing *Foeniculum vulgare* (fennel);
2. A consumer with a history of heart disease described an adverse effect (dizziness) due to a PFS containing *Paullinia cupana* (guarana);
3. A consumer reported an unspecified adverse effect due to a PFS containing *Aloe barbadensis* (aloe) and *Harpagophytum procumbens* (devil's claw);
4. A consumer experienced tachycardia after the intake of a PFS containing *Panax ginseng* (ginseng);
5. A consumer reported discomfort (mainly nausea) associated with a PFS containing *Cyamopsis tetragonoloba* (guar).

### The 20 most frequently used botanicals

Table 8 lists the botanicals most frequently used in Europe (6 countries), Italy and the 4 Italian cities; botanicals in the three first positions are highlighted (grey cells) in each list. In Italy, the botanicals in the three first positions are: 1) *Aloe vera* (aloe); 2) *Foeniculum vulgare* (fennel); and *Valeriana officinalis* (valerian). All of them are among the European top 20 botanicals, but in lower position (5, 6 and 7, respectively). In the four Italian cities:

1. Aloe is always in the first position;
2. Fennel is in the second position everywhere apart from Catania, where the second most used botanical is valerian;
3. Different botanicals are placed in the third position: ginseng and *Passiflora incarnata* (purple passion flowers) for Milan; *Vaccinium myrtillus* (blueberry) for Venice and Rome; and ginseng for Catania.

**Table 8:** The top 20 plants reported for consumption in Italy, overall and by city, and comparison with the whole survey^a^^,^^b^^,^^c^.

			EUROPE^d^		ITALY		MILAN		VENICE		ROME		CATANIA	
	Latin name	Common name	n	%	n	%	n	%	n	%	n	%	n	%
**1**	*Aloe vera (L*.*)* Burm.f.	Aloe vera	145	6.2	44	11.6	10	10.4	7	7.8	14	14.6	13	13.5
**2**	*Foeniculum vulgare* Mill	Fennel	132	5.6	29	7.7	10	10.4	7	7.8	8	8.3	4	4.2
**3**	*Valeriana officinalis* L.	Valerian	125	5.3	29	7.7	6	6.3	5	5.6	5	5.2	13	13.5
**4**	*Panax ginseng* C.A. Mey	Ginseng	167	7.1	28	7.4	8	8.3	4	4.4	4	4.2	12	12.5
**5**	*Vaccinium myrtillus* L.	Blueberry	100	4.2	28	7.4	6	6.3	7	7.8	10	10.4	5	5.2
**6**	*Passiflora incarnata* L.	Purple passion flower	78	3.3	26	6.9	8	8.3	5	5.6	7	7.3	6	6.3
**7**	*Melissa officinalis* L.	Lemon balm	103	4.4	25	6.6	7	7.3	5	5.6	6	6.3	7	7.3
**8**	*Paullinia cupana* Kunth	Guarana	72	3.1	23	6.1	6	6.3	5	5.6	3	3.1	9	9.4
**9**	*Taraxacum officinale* F.H. Wigg	Dandelion	80	3.4	21	5.6	7	7.3	9	10.0	4	4.2	1	1.0
**10**	*Cynara scolymus* L.	Globe artichoke	173	7.3	20	5.3	6	6.3	5	5.6	8	8.3	1	1.0
**11**	*Senna alexandrina* Mill.	Alexandrian senna	60	2.5	19	5.0	5	5.2	7	7.8	3	3.1	4	4.2
**12**	*Ginkgo biloba* L.	Ginkgo	194	8.2	17	4.5	1	1.0	4	4.4	8	8.3	4	4.2
**13**	*Centella asiatica* (L.) Urb.	Spadeleaf	19	0.8	15	4.0	4	4.2	3	3.3	7	7.3	1	1.0
**14**	*Rosa canina* L.	Dog rose	44	1.9	15	4.0	5	5.2	2	2.2	4	4.2	4	4.2
**15**	*Silybum marianum* (L.) Gaertn.	Milk thisle	69	2.9	15	4.0	7	7.3	5	5.6	1	1.0	2	2.1
**16**	*Eleutherococcus senticosus* (Rupr. & Maxim.) Maxim	Siberian ginseng	28	1.2	14	3.7	4	4.2	4	4.4	2	2.1	4	4.2
**17**	*Glycyrrhiza glabra* L.	Licorice	74	3.1	14	3.7	1	1.0	5	5.6	5	5.2	3	3.1
**18**	*Malpighia glabra* L.	Wild crapemyrtle	71	3.0	14	3.7	3	3.1	5	5.6	1	1.0	4	4.2
**19**	*Cuminum cyminum* L.	Cumin	14	0.6	13	3.4	3	3.1	6	6.7	4	4.2	0	0
**20**	*Harpagophytum procumbens* (Burch.) DC. ex Meisn	Devil's claw	75	3.2	13	3.4	3	3.1	2	2.2	6	6.3	2	2.1
			*n = 2359*		*n = 378*		*n = 96*		*n = 90*		*n = 96*		*n = 96*	

^a^grey cells indicate the botanicals in the three first positions

^b^since PFS can contain more than one ingredient, the total counts are higher than the total PFS cited; percentage are referred to the total consumers' samples

^c^top plants are listed according to the decreasing order of reply coming from the Italian respondents

^d^in the whole survey the three most cited plants were: 1) *Ginkgo biloba* L.; 2) *Oenothera biennis* L. (194, not present in the top Italian list); and 3) *Cynara scolymus* L.

### Consumers’ habits

Analysis of the Italian consumers' habits by sex or age group (Table 9) reveals some interesting differences:

1. Females showed the same pattern of preferences of the whole Italian sample with an exception in the third position in favour of blueberry;
2. Males showed a preference for PFS containing, in decreasing order: aloe, ginseng and *Melissa officinalis* (lemon balm);
3. Younger consumers ranked ginseng in third position;
4. Consumers aged over 60 years cited purple passion flowers in the third position.

**Table 9:** The top 20 consumed plants in Italy by sex and age^a^^,^^b^.

			ITALY		MALE		FEMALE		18–59 yrs		> 60 yrs	
	Latin name	Common name	n	%	n	%	n	%	n	%	n	%
**1**	*Aloe vera (L*.*)* Burm.f.	Aloe vera	44	11.6	27	14.4	17	8.9	24	8.5	20	21.3
**2**	*Foeniculum vulgare* Mill	Fennel	29	7.7	12	6.4	17	8.9	20	7.0	9	9.6
**3**	*Valeriana officinalis* L.	Valerian	29	7.7	14	7.5	15	7.9	25	8.8	4	4.3
**4**	*Panax ginseng* C.A. Mey	Ginseng	28	7.4	20	10.7	8	4.2	24	8.5	4	4.3
**5**	*Vaccinium myrtillus* L.	Blueberry	28	7.4	13	7.0	15	7.9	23	8.1	5	5.3
**6**	*Passiflora incarnata* L.	Purple passion flower	26	6.9	12	6.4	14	7.3	19	6.7	7	7.4
**7**	*Melissa officinalis* L.	Lemon balm	25	6.6	16	8.6	9	4.7	23	8.1	2	2.1
**8**	*Paullinia cupana* Kunth	Guarana	23	6.1	14	7.5	9	4.7	20	7.0	3	3.2
**9**	*Taraxacum officinale* F.H. Wigg	Dandelion	21	5.6	9	4.8	12	6.3	19	6.7	2	2.1
**10**	*Cynara scolymus* L.	Globe artichoke	20	5.3	9	4.8	11	5.6	14	4.9	6	6.4
**11**	*Senna alexandrina* Mill.	Alexandrian senna	19	5.0	9	4.8	10	5.2	14	4.9	5	5.3
**12**	*Ginkgo biloba* L.	Ginkgo	17	4.5	8	4.3	9	4.7	12	4.2	5	5.3
**13**	*Centella asiatica* (L.) Urb.	Spadeleaf	15	4.0	7	3.7	8	4.2	12	4.2	3	3.2
**14**	*Rosa canina* L.	Dog rose	15	4.0	5	2.7	10	5.2	11	3.9	4	4.3
**15**	*Silybum marianum* (L.) Gaertn.	Milk thisle	15	4.0	11	5.9	4	2.1	8	2.8	7	7.4
**16**	*Eleutherococcus senticosus* (Rupr. & Maxim.) Maxim	Siberian ginseng	14	3.7	11	5.9	3	1.6	12	4.2	2	2.1
**17**	*Glycyrrhiza glabra* L.	Licorice	14	3.7	4	2.1	10	5.2	10	3.5	4	4.3
**18**	*Malpighia glabra* L.	Wild crapemyrtle	14	3.7	9	4.8	5	2.6	13	4.6	1	1.1
**19**	*Cuminum cyminum* L.	Cumin	13	3.4	5	2.7	8	4.2	10	3.5	3	3.2
**20**	*Harpagophytum procumbens* (Burch.) DC. ex Meisn	Devil's claw	13	3.4	6	3.2	7	3.7	6	2.1	7	7.4
	***Total consumers' samples***		*n = 378*		*n = 187*		*n = 191*		*n = 284*		*n = 94*	

^a^grey cells indicate the botanicals in the three first positions

^b^since PFS can contain more than one ingredient, the total counts are higher than the total PFS referred; percentages refers to the total consumers' samples

## Discussion

One of the most debated topics about PFS is their actual intake by consumers; reliable available data are relatively few [10–12], limited to a specific country [13–14], and usually include all types of food supplements [10, 15–17]. In a study on the use of food supplements in Italy published by Giammarioli et al [18], data were collected sending a questionnaire by mail to 10 000 Italian citizens. According to the 1722 questionnaires received back, vitamin and/or mineral supplements were the most used (61%), followed by PFS (28%).

A paper by European Advisory Service—EAS [19] reported information on the European market and its regulation and evidenced the need for new data in order to plan, monitor and evaluate PFS intake with the objective of assessing their benefits and risks. The European project PlantLIBRA defined, as one of its most important aims, the collection of information to fill this gap. Data from the whole survey has been published previously [7]; this paper analyses in more detail the situation in Italy, one of the six countries involved in the PlantLIBRA PFS consumers' survey. In some ways, the survey in Italy was easier than in other European countries since most products containing botanicals are classified as food supplements, and are very rarely associated with the traditional or other alternative medicines (CAM). In other words, Italian consumers use PFS for improving their health, sometimes in the hope of obtaining a specific beneficial activity (e.g. on hypercholesterolemia, heart diseases, immune disorders)

In Italy, the interest for PFS (food supplements containing botanicals) is high, in fact:

1. In the PlantLIBRA Italian sample, the calculated weighted prevalence of "regular" PFS consumers was 22.7%, indicating that approximately one out of four Italians uses PFS during the year (periodically or when there is a worsening of the health status);
2. The number of products taken by 387 Italian consumers was 289.

Among Italian consumers, some differences were observed when four selected cities were considered separately:

1. The pattern of use during the year is specific for each city, with opposite trends for example in July;
2. Milan consumers reported reasons of use significantly different from those of the whole Italian sample and did not indicate supermarkets as an important place of purchase;
3. Respondents from Rome and Catania more frequently used family doctors and pharmacists as a source of recommendation;
4. Significant differences among cities, sex and age groups were observed when the botanicals were ranked in order of frequency of use.

## Conclusions

Data from this paper provides new insights on the socio-economic characteristics and lifestyle of Italian PFS consumers, on the reasons and pattern of use, and finally details on their behaviour and expectations. New information was collected on the frequency of use of botanicals, including the specific pattern of 4 major Italian cities. Even though it is difficult to estimate the actual dose of PFS/botanical ingredient consumed (due to the limited information included in the labelling), new data are now at our disposal for future discussion and assessment on the risk and benefits associated with the increasing use of PFS.
